# Central Venous Catheter-Associated Pericardial Tamponade in a 6-Day Old: A Case Report

**DOI:** 10.1155/2009/910208

**Published:** 2010-02-09

**Authors:** Swati O. Arya, Gurumurthy M. Hiremath, Kingsley C. Okonkwo, Michael D. Pettersen

**Affiliations:** The Carman and Ann Adams Department of Pediatrics, Wayne State University and Division of Pediatric Cardiology, Children's Hospital of Michigan, Detroit, MI 48201, USA

## Abstract

*Introduction*. Pericardial effusion (PCE) and tamponade can cause significant morbidity and mortality in neonates. Such cases have been reported in the literature in various contexts. *Case Presentation*. A 6-day old neonate with meconium aspiration syndrome and persistent pulmonary hypertension of newborn on high frequency oscillator ventilation and inhaled nitric oxide was referred to our hospital with a large pericardial effusion causing hemodynamic compromise. Prompt pericardiocentesis led to significant improvement in the cardio-respiratory status and removal of the central line prevented the fluid from reaccumulating. Cellular and biochemical analysis aided in the diagnosis of catheter related etiology with possibility of infusate diffusion into the pericardial space. *Conclusion*. We present this paper to emphasize the importance of recognizing this uncommon but serious complication of central venous catheters in intensive care units. We also discuss the proposed hypothesis for the mechanism of production of PCE.

## 1. Introduction

Pericardial effusion (PCE) and resulting tamponade are rare but often fatal complications associated with central venous catheters (CVCs). They account for up to 0.7% of central venous catheter-associated complications [1, 2]. Pericardial effusions have been reported at any time from the insertion of the catheter to 112 days later, with the median time to occurrence being 3 days after insertion [1, 2]. Although rare, it is vital to recognize this complication of central lines in neonates since failure to do so can result in significant morbidity and mortality, especially in low birth weight babies. The purpose of this paper is to emphasize the occurrence of CVC-related cardiac complications and to urge clinicians to keep a high index of suspicion for pericardial effusion in neonates with central lines, who acutely deteriorate or have unexplained instability in cardiopulmonary status. We will also review the proposed hypothesis for the mechanism of CVC-associated PCE and tamponade.

## 2. Case Presentation

A full term neonate born to a G2P2 mother by C-section (performed for nonreassuring fetal status). At delivery, the amniotic fluid was found to be meconium stained. The infant demonstrated decreased tone and poor respiratory effort requiring intubation and aspiration of meconium. APGAR score was 3, 7, and 8 at 1, 5, and 10 minutes, respectively; birth weight was 2520 grams. He developed meconium aspiration syndrome (MAS) and resultant persistent pulmonary hypertension of the newborn (PPHN) and was placed on a conventional ventilator. Due to worsening respiratory status, he was later placed on high frequency oscillator ventilator (HFOV) with subsequent addition of inhaled nitric oxide on Day 6 of life. The baby required inotropic support in the form of Dopamine, Dobutamine, and Hydrocortisone from Day 1. As a complication of MAS and its treatment, he developed bilateral pneumothoraces, for which a chest tube was placed in the right pleural space. A double lumen umbilical venous catheter (UVC) was placed and total parenteral nutrition started on Day 2 of life at 6.4 cc/hr; 20% intralipid solution was added the next day at 1.6 cc/hr. We also found that the TPN was transfused through the proximal lumen of the UVC. Due to continued cardio-respiratory instability, an echocardiogram was performed on day 6 of life. This study demonstrated a large pericardial effusion prompting transfer to our institution. The study did not show any malposition of the catheter. 

On arrival baby was on HFOV, Dopamine, and Dobutamine at 15 mcg/kg/min. Vitals on admission were as follows: T:37.0 C, Heart rate:160/min, BP:79/55, MAP:63 mmHg, pulse-oximetry was 90%. We did not measure CVP on the infant. Weight on admission was 2645 grams. CXR on admission showed mild enlargement of cardio-thymic silhouette, right pleural effusion, and tip of the UVC just inferior to the diaphragm. All chest X-rays done from day 1 including the one done at our institution showed satisfactory position of UVC tip. Repeat echocardiogram confirmed the presence of a large global pericardial effusion. Cardiac anatomy and ventricular function were normal. Pericardiocentesis was performed under echocardiographic guidance. The effusion was incompletely evacuated with removal of 9 mL of milky pericardial fluid which was sent for analysis. We were unable to remove more fluid form the pericardial cavity in that setting raising the possibility that a relatively small amount of fluid may have collected in the pericardial space in a short duration of time. All infusions through the UVC were stopped.

Postpericardiocentesis, there was improvement in the hemodynamic status of the neonate. The heart rate decreased by 30–40 beats/min, the systolic blood pressure improved by 10 mmHg, and oxygen saturation improved to >95%. Thus, the hemodynamic compromise caused by the cardiac tamponade was taken care of and the infant's cardio-respiratory status improved to some extent. The infant stayed on high frequency ventilator for underlying PPHN for few more days. Analysis of pericardial fluid revealed glucose was 252, triglycerides, 156, 1 nucleated cell, and 1500 RBCs. Fluid culture failed to grow any organism in the next 4 days. Pleural fluid was aspirated the next day for chemistry and cell count. The fluid was bloody in appearance with 14000 red blood cells and 720 nucleated cells; predominantly neutrophils. Glucose was 300, protein <2, triglycerides 23, and LDH of 151. Hence it was difficult to comment on the nature and origin of the pleural fluid.

Repeat echocardiogram performed the following day revealed a moderate, predominantly anterior pericardial effusion. Some centers perform a dye test to confirm the extravasation but this test is not done routinely at our center. The UVC was removed once the results of the fluid biochemistry were received and the infant received a new peripherally inserted central catheter line. On hospital day 3, a small anterior pericardial effusion was present and on day 10, the effusion had completely resolved. The infant was eventually weaned to conventional ventilator and ultimately extubated.

## 3. Discussion

PCE and tamponade are known complications of CVCs. With the increased use of long-term CVCs in neonatal intensive care units, there has been an increase in the incidence of PCE associated with total parenteral nutrition.

The etiology of such a pericardial effusion is not clear; however several possibilities have been proposed based on clinical and autopsy findings [1–3]. The myocardium in neonates may have areas of weakness which may be vulnerable to injury since it is not completely muscularized [2]. Repeated contact of the catheter tip with the cardiac wall with each contraction leads to endothelial cell damage and subsequent adherence of platelets and activation of the coagulation cascade. The resulting thrombus fosters attachment of the catheter tip to the heart, causing irritation of the endothelial cell lining by the infusate, causing osmotic injury. Through the damaged lining, fluid then diffuses into the pericardial space forming an effusion. In our case, we did not appreciate any thrombus at the catheter tip on echocardiogram or on removal. Depending on the acuity and severity of the diffusion, cardiac tamponade and even overt myocardial perforation can occur. In instances where the TPN is infused through the proximal port of the UVC, deposits of lipids placed inside the liver may possibly diffuse to the pericardium via collaterals. The termination site of the catheter and the angle of the catheter within the heart may contribute to injury, for example, loops or curves in the device are associated with a greater incidence of myocardial perforation [4]. Interestingly, a case has been reported by Onal et al. from Turkey describing a term infant developing tamponade despite correct position of the UVC [5]. Hyperosmolar infusates causing endothelial damage and transmural necrosis seems to be the mechanism of effusion in these cases. Based on their experience, they suggest that high index of suspicion be maintained despite satisfactory position of the catheter when an infant with a central line deteriorates hemodynamically. Another such report has been described by Sehgal et al. with a properly positioned UVC [6].

A retrospective review of pericardial effusions attributed to central venous catheters (which includes UVCs) (*n* = 61) by Nowlen et al. revealed that 92% of all catheters were last reported to be within the pericardial silhouette (82% were in the heart and 10% were at the vena cava/right atrial junction) at the time the pericardial effusion was detected [1]. We did not find similar data pertaining to UVCs only, except individual case reports [5, 6]. The biochemical analysis of pericardial fluid in our case with high glucose and increased triglycerides in the absence of cells supports CVC-related fluid collection with TPN diffusion through myocardium. The RBCs in the fluid likely resulted from the procedure itself. During pericardiocentesis, the fluid was initially milky and became blood tinged after repeat attempts to evacuate the fluid. Review of literature suggests that most cases of the pericardial effusion resulting from central lines do not show evidence of trauma, supporting the hypothesis that there is osmotic diffusion of infusate even before overt myocardial perforation occurs [1].

Nonspecific signs of PCE/tamponade include muffled or absent heart sounds, tachycardia or bradycardia, weak peripheral pulses, pallor, cyanosis, poor perfusion, increasing inotropic support, or unexplained deterioration of cardiopulmonary status [1, 7, 8].

There is considerable debate regarding the type and material of the central lines being used and correct tip placement of a CVC. Studies in vitro and in adults suggest that thin, flexible silastic catheters are less likely to perforate and an increased angle of incidence between the CVC tip and the cardiac/vessel wall increases the likelihood of perforation [9, 10]. Hence it is recommended that routine radiography be performed on patients with CVC tips near the heart to ensure that the tip has not migrated. The CVC tip should remain outside the cardiac silhouette but still within the vena cavae. Tip position in the high superior vena cavae or below the inferior vena cavae/right atrial junction should keep the CVC outside the pericardial reflections and thus minimize the risk of perforation. Studies have also suggested that the accuracy of chest radiography is suboptimal in predicting catheter position. In a study by Ades et al., the sensitivity and specificity of cheat radiography in evaluating inappropriate catheter position were 32% and 89% [11]. Therefore, at the slightest suspicion of such a complication, prompt evaluation with ultrasound of the abdomen and heart must be performed to save valuable time. 

For management of such PCE/tamponade, stopping all infusions through the CVC is recommended followed by aspiration from the catheter/PICC as a primary tool for evacuation of the effusion. We did not attempt to aspirate in our case since the UVC tip position was satisfactory at the time we evaluated the infant and so aspiration of fluid through UVC was not expected in our case. If symptoms persist, pericardiocentesis should be performed. The central line may need to be repositioned or replaced [1].

## 4. Conclusion

PCE/tamponade is a rare but serious complication seen in neonates with CVCs which can happen even with correct position of the catheter tip. A high index of suspicion should be maintained in neonates with hemodynamic and respiratory instability and early echocardiography should be performed. Measures for prevention include routine radiography and identification of CVC tip. The CVC tip should remain outside the cardiac silhouette but still within the vena cavae. Catheter should be regularly checked for tip migration. We think there should be a low threshold of performing an echocardiogram in sick neonates with central lines and may be repeated if there is any deterioration in clinical status. Pericardiocentesis may be life saving procedure in these cases and leads to immediate hemodynamic improvement in addition to aiding in the diagnosis. Cellular and biochemical analysis of the fluid also helps rule out other causes of pericardial effusion such as infections.

##  Consent

Written informed consent was obtained from the patient for publication of this case report and accompanying images. A copy of the written consent is available for review by the Editor-in-Chief of this journal.

##  Competing Interests

The authors declare that they have no competing interests.

## Figures and Tables

**Figure 1:**
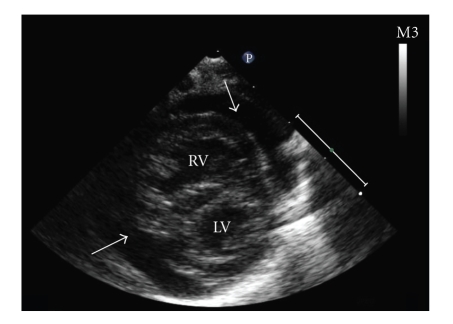
Echocardiogram on day 1 of admission showing massive pericardial effusion present globally around the heart.

## Abbreviations

CVC: Central venous catheter
PCE: Pericardial effusion
RBC: Red blood cell
HFOV: High frequency oscillator ventilation
MAS: Meconium aspiration syndrome
PPHN: Persistent pulmonary hypertension of the newborn.
